# Recent trends in extraction, purification, structural characterization, and biological activities evaluation of *Perilla frutescens* (L.) Britton polysaccharide

**DOI:** 10.3389/fnut.2024.1359813

**Published:** 2024-02-22

**Authors:** Ling Zhu, Lijun Guan, Kunlun Wang, Chuanying Ren, Yang Gao, Jialei Li, Song Yan, Xindi Zhang, Xinmiao Yao, Ye Zhou, Bo Li, Shuwen Lu

**Affiliations:** ^1^Institute of Food Processing, Heilongjiang Province Academy of Agricultural Sciences, Harbin, China; ^2^Heilongjiang Province Key Laboratory of Food Processing, Harbin, China

**Keywords:** *Perilla frutescens*, polysaccharides, extraction, structural characterization, bioactivities

## Abstract

*Perilla frutescens* (L.) Britton is an annual herb plant of the *Perilla* genus in the Labiatae family, which is commonly utilized as an edible and medicinal resource. Polysaccharides are among the major components and essential bioactive compounds of *P. frutescens*, which exhibit a multitude of biological activities, including antioxidant, antitumor, anti-fatigue, immunoregulation, hepatoprotective, anti-inflammatory, and lipid-lowering effects. As a natural carbohydrate, *P. frutescens* polysaccharide has the potential to be utilized in the development of drugs and functional materials. In this paper, we provide an overview of progress made on the extraction, purification, structural characterization, and bioactivity of polysaccharides from different parts of *P. frutescens*. The challenges and opportunities for research are discussed, along with the potential development prospects and future areas of focus in the study of *P. frutescens* polysaccharides.

## 1 Introduction

**Figure 1:**
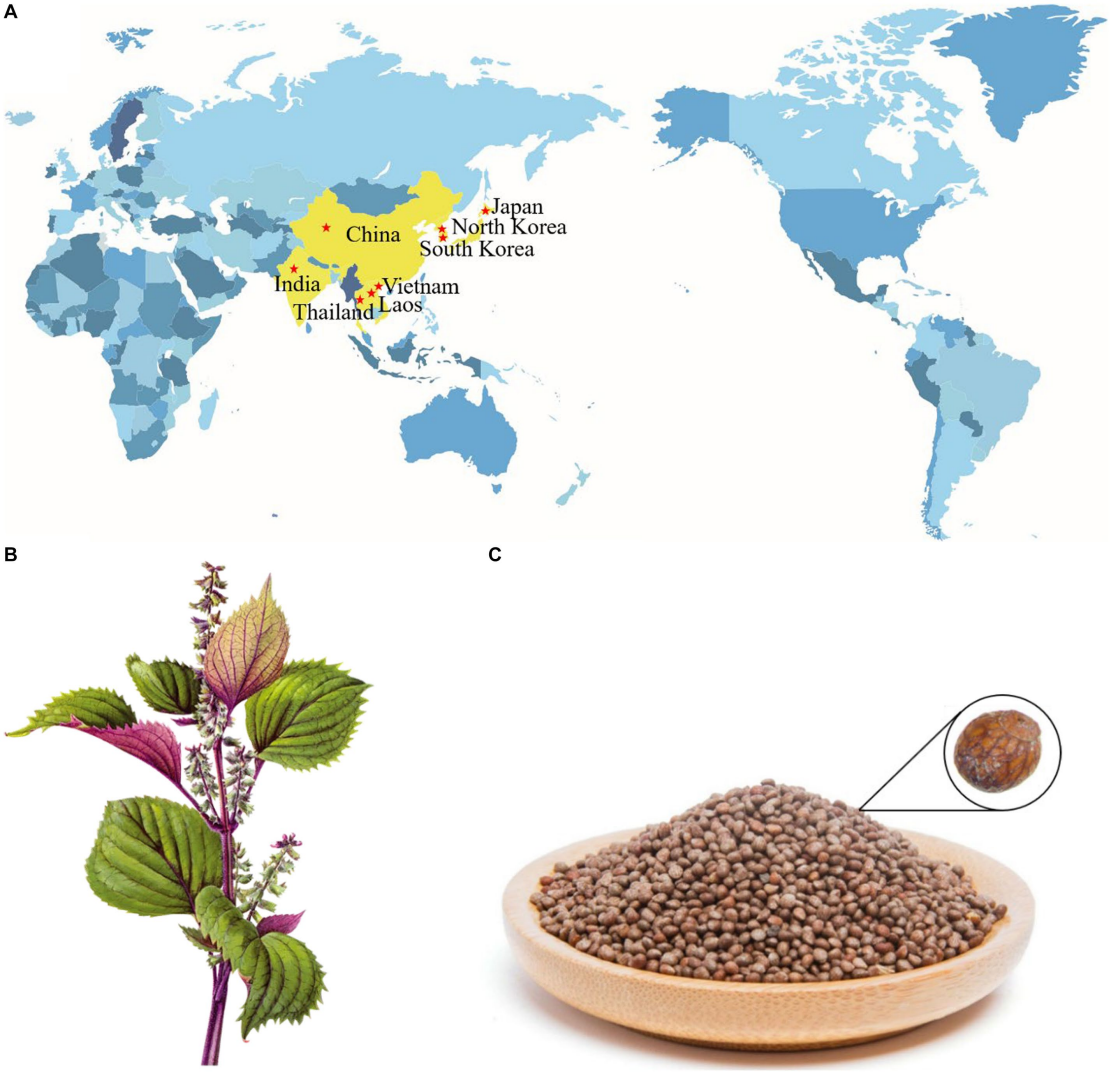
Distribution diagram of *P. frutescens* in the world **(A)**; stems and leaves of *P. frutescens* **(B)**; seeds of *P. frutescens* **(C)**.

*Perilla frutescens* (L.) Britton, also known as zisu in China, is an annual herb plant of the Labiatae family (1, 2). Although *P. frutescens* is widespread in Asian countries (Figure 1A) (3, 4), China is proposed to be the main genetic center of this species, where it has been cultivated for more than 2,000 years (5, 6). According to the variation of plant leaf color, *P. frutescens* can be divided into two main varieties circulating in China: *P. frutescens* var. *arguta* (the lower part of the leaf is red or purple) and *P. frutescens* var. *frutescens* (the upper and lower leaf surfaces are green) (7, 8). *P. frutescens* is considered a medicine and food homologous plant, as the dried stems, leaves (Figure 1B), and seeds (Figure 1C) can be used as a natural herbal medicine for pain relief, hemostasis, relief of cough, purgative, detoxification, relief of stomach upset, dissipating colds, and anti-inflammation (8–10). As part of the daily diet, *P. frutescens* leaves can be used in barbecue, sashimi, sushi, and as a culinary condiment given their aromatic flavor (9, 11). Additionally, the oil of *P. frutescens* seeds can be used in baking pastries as an alternative to hydrogenated oils or cream (9). *P. frutescens* is becoming a more popular item in home cooking as it is increasingly recognized as a health-promoting food source.

In recent years, the chemical composition of *P. frutescens* has been extensively investigated (12). *P. frutescens* contains multiple active ingredients, including polysaccharides, anthocyanins, flavonoids, terpenoids, phenols, volatile oils, fatty acids, and proteins (13, 14). Among these compounds, polysaccharide is an essential component, which has been increasingly researched for various applications owing to its advantages of natural origin, safety, and low toxicity (15). The polysaccharides obtained from *P. frutescens* have proven to exhibit antioxidant, hepatoprotective, antitumor, and immunomodulatory activities (16–19), highlighting their broad application prospects in the food and biomedicine industries. To date, studies in this field have mainly focused on the small-molecule compounds of *P. frutescens* and their pharmacological effects; however, a comprehensive review of the macromolecular polysaccharides of *P. frutescens* is lacking. Hence, in the present paper, the extraction, purification, structural characteristics, and biological activities of *P. frutescens* polysaccharides are systematically summarized with a view to further expand their application areas.

## 2 Extraction and purification of polysaccharides from *Perilla frutescens*

### 2.1 Extraction of polysaccharides from *Perilla frutescens*

The extraction of polysaccharides from raw materials is the first and key step in their effective utilization. Therefore, identifying optimal extraction methods has been a primary research focus. *P. frutescens* polysaccharides are mainly extracted from the leaf and seed meal (a by-product of the seed after oil extraction) of the plant. Typically, a suitable solvent (such as petroleum ether, butane, or *n*-hexane) is selected to degrease the *P. frutescens* raw material in a Soxhlet extractor prior to polysaccharide extraction (15, 18, 20). Polysaccharides are a class of highly polar, highly water-soluble, and ethanol-insoluble substances that can be obtained by hot water extraction (HWE) in combination with ethanol precipitation (21). For instance, Zhou and Sheng (22) prepared polysaccharides from *P. frutescens* seeds using HWE at 80 °C followed by 70% ethanol alcohol precipitation. To increase the polysaccharide yield, response surface methodology (RSM) can be used to optimize the extraction conditions. For example, Ding (23) determined the following optimized HWE conditions for the extraction of *P. frutescens* leaf polysaccharide using RSM: material-to-liquid ratio of 1:30, extraction time of 4 h, extraction three times, and ethanol concentration for precipitation of 60% (*v/v*). Under the above conditions, the extraction yield of crude *P. frutescens* leaf polysaccharide reached up to 5.22 ± 0.17%. HWE has advantages of being a relatively simple, low-cost, and non-polluting process (24); however, this method suffers from the drawbacks of a long operating time, low yield, and the need for repetitive operations (25). Thus, HWE should be combined with other innovative technologies such as ultrasonic-assisted extraction (UAE), microwave-assisted extraction (MAE), and ultrasonic-assisted enzyme extraction (UAEE).

UAE can promote the release and dissolution of intracellular and cell wall polysaccharides through the high shear pressure generated via cavitation, which has the benefits of a high extraction rate, simple operation, and low solvent dosage (26). Zhang et al. (18) optimized the experimental scheme for the extraction of polysaccharides from *P. frutescens* seed meal (PSMP) by UAE through RSM, obtaining an average extraction yield of 6.137 ± 0.062% using a liquid-to-solid ratio of 26.00 mL/g, extraction temperature of 43.00°C, ultrasonic time of 52.00 min, and ultrasonic power of 229.00 W. Compared with the time required using HWE, the extraction time for obtaining *P. frutescens* polysaccharides could be shortened by two-thirds and the extraction temperature was reduced by 33.70–57.40% with UAE (20).

MAE has the advantages of high permeability, selectivity, and extraction efficiency (27), demonstrating its suitability for the extraction of *P. frutescens* polysaccharide. Microwave is an electromagnetic wave with a frequency in the range of 300 MHz to 300 GHz (24). Microwave heating is produced by the ionic conduction of dissolved ions and molecular friction due to the dipolar rotation of polar solvents (28). The optimized conditions for the MAE of *P. frutescens* seed polysaccharides were determined to be a liquid-to-feed ratio of 25 mL/g, microwave power of 480 W, and microwave processing time of 3 min, which resulted in a yield of 9.06% with subsequent HWE (90°C, 3 h) (29). In another study, using the MAE method to extract polysaccharides from *P. frutescens* leaf powder with a short microwave treatment (800 W) of only 30 s after hot water immersion (80°C) for 2 h resulted in improvement of the yield to 3.99% compared to a yield of only 2.21% obtained with the HWE method, which required a total extraction time of 6 h at 80°C (30). Therefore, the extraction of *P. frutescens* polysaccharides by MAE can save substantial time while improving the efficiency compared with the HWE method.

Enzyme-assisted extraction of polysaccharides involves the use of enzymes capable of breaking down the cell wall, which offers benefits of environmental friendliness, low-energy consumption, and high extraction efficiency (31, 32). Under the optimal conditions of 1771.85 U/g cellulase, enzyme activation temperature of 53.7°C, and enzyme digestion time of 36.2 min, the extraction rate of *P. frutescens* leaf polysaccharides reached 17.91 mg/g (33). Ultrasonic extraction can further enhance the affinity of the enzyme for the substrate and thereby increase the speed of the enzymatic reaction (24). Therefore, enzyme-assisted extraction can be combined with UAE (i.e., UAEE) to effectively increase the extraction rate. Recently, Li et al. (34) optimized the UAEE process to extract polysaccharides from *P. frutescens* leaves according to a single-factor test and Box-Behnken design (BBD), obtaining a yield of 3.84% when the liquid-to-solid ratio was 41:1, enzymatic time was 40 min, enzymatic temperature was 49 °C, and ultrasonic power was 204 W. Subsequently, Zhang et al. (35) applied RSM analysis with a BBD to identify the optimal conditions for the UAEE of *P. frutescens* seed meal polysaccharides (PSMP). With the optimized conditions of a compound enzyme dose of 6.6%, liquid-to-solid ratio of 25 mL/g, extraction time of 61 min, and extraction temperature of 62°C, the PSMP yield was 7.711 ± 0.201%.

In addition to the methods described above, various novel approaches for extracting polysaccharides have also been proposed, including pressurized-liquid extraction, supercritical-fluid extraction, ionic-liquid extraction, and pulsed electric field-assisted extraction (27, 32). However, these novel approaches have not yet been employed for extracting polysaccharides from *P. frutescens*, requiring further investigation in the future.

### 2.2 Purification of polysaccharides from *Perilla frutescens*

The crude polysaccharide obtained after ethanol precipitation normally contains impurities such as proteins, pigments, and small molecules, necessitating subsequent separation and purification processes to obtain the pure polysaccharide (36). The Sevag method is commonly used to remove proteins from crude polysaccharide solutions, which is based on the principle of protein denaturation in chloroform and other organic solvents (17). In brief, the crude polysaccharide solution is blended with Sevag’s reagent (1-butanol/chloroform, 1/4 *v/v*) at a certain proportion, the mixture is shaken well, and the supernatant is collected after centrifugation to remove some of the proteins (37, 38). The Sevag method offers advantages of mild conditions, without causing the denaturation of polysaccharides (39), and can achieve a good effect of removing proteins from the crude *P. frutescens* polysaccharide solution after multiple repeated operations (20, 23, 34). Zhang (30) discovered that under the conditions of a 4:1 ratio of chloroform to n-butanol, 1:1 ratio of polysaccharide solution to Sevag reagent, 35 min of deproteinization time, and two deproteinization steps, the deproteinization rate of *P. frutescens* leaf polysaccharide reached 73.6%. However, the deproteinized *P. frutescens* polysaccharide solution needs to be further dialyzed to remove residual Sevag reagent and other small-molecule impurities (15, 35).

To obtain homogeneous polysaccharides, further isolation and purification of the crude polysaccharides is required. Column chromatography is the most extensively applied approach for the purification of polysaccharides owing to its good purification effect and simple operation procedures (40–42). Column chromatography mainly includes ion-exchange column chromatography and gel-column chromatography (43). Ion-exchange chromatography involves the use of either cation- or anion-exchange resins (44), with anionic columns most frequently used in the purification of *P. frutescens* polysaccharides. Anion-exchange chromatography enables strong binding to acidic polysaccharides without interacting with neutral polysaccharides, thus, the neutral polysaccharides are eluted first (45) and those obtained from different fractions can be separated by a stepwise elution process using solutions with different ionic strengths (46). For example, Li et al. (34) eluted four types of *P. frutescens* leaf polysaccharides on a DAE-Cellulose 52 column (2.6 × 30 cm) at a flow rate of 0.6 mL/min using different concentrations of sodium chloride solution (0, 0.1, 0.2, 0.3, and 0.5 mol/L).

**Figure 2:**
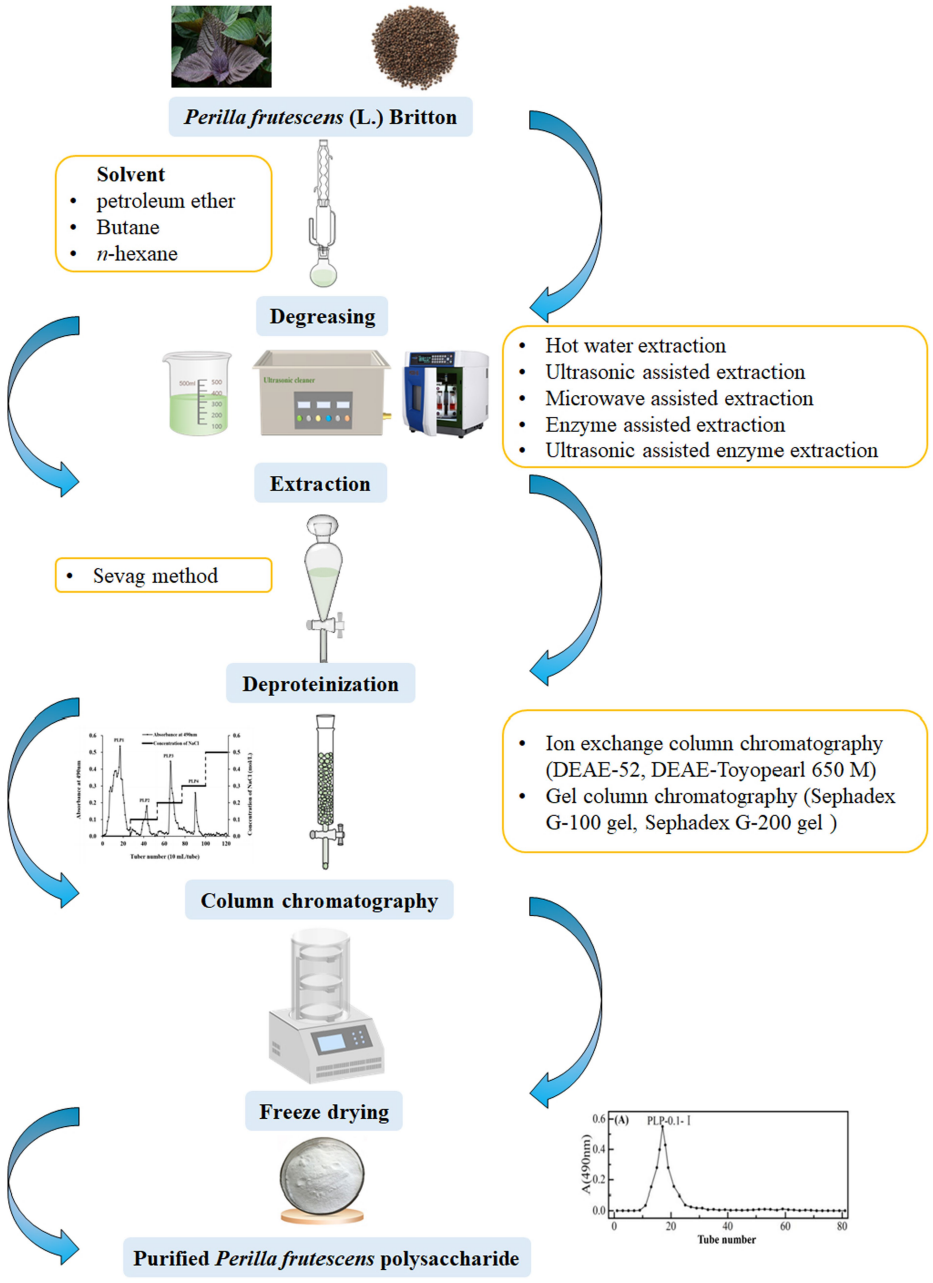
Schematic diagram of the extraction and purification process of *P. frutescens* polysaccharide.

Gel-column chromatography (Sephadex-G series and Sephacryl-S series) is an effective method to separate polysaccharides with different molecular weights (47). High-molecular-weight polysaccharides will be eluted first with the mobile phase, while the low-molecular-weight polysaccharides will diffuse into the pores of the gel and elute later (48). Ding et al. (15) used a Sephadex G-200 gel column (1.6 cm × 30 cm) to purify and obtain a *P. frutescens* leaf polysaccharide (PFP) with purity reaching up to 89.73%. Different columns can be used synergistically in the separation and purification of polysaccharides to achieve a better effect. Kim et al. (16) first used a DEAE-Toyopearl 650 M column (4.0 × 30 cm) to isolate one polysaccharide, designated PFB-1-0. This polysaccharide was then eluted using a Sephadex G-100 gel column (2.5 × 94 cm) with 0.2 mol/L NaCl at a flow rate of 0.2 mL/min to obtain fractions PFB-1-0i and PFB-1-0ii with 43.2 ± 2.5% and 82.8 ± 4.8% purity, respectively. In the majority of studies, DEAE-52 cellulose, DEAE-Toyopearl 650 M, Sephadex G-100 gel, and Sephadex G-200 gel chromatography columns are employed for the separation of *P. frutescens* polysaccharides, the eluate is collected, concentrated, and freeze-dried to finally obtain purified *P. frutescens* polysaccharides. The flow chart of the extraction and purification procedure of *P. frutescens* polysaccharides is displayed in Table 1 and Figure 2.

**Table 1:** Extraction and purification of polysaccharides from the different parts of *P. frutescens*.

Polysaccharide name	Source	Extraction method	Crude polysaccharide yield	Separation and purification progress	References
PFP	Leaf	Petroleum ether, degreasing, HWE, solid to liquid ratio of 1:30, 100°C, 3 replicate extractions, EtOH precipitation	5.22 ± 0.17%	Water dialysis for 2 days, Sephadex G-200 gel column chromatography	(15)
PFB-1-0-ii	Leaf	HWE, 20 mL/g liquid-solid ratio, 100°C, methanol reflux, 80% EtOH precipitation	9.8%	DEAE-Toyopearl 650 M, Sephadex G-100 column chromatography	(16)
PEPF	Leaf	HWE, 10 mL/g liquid-solid ratio, 90°C, 2 h, 70% EtOH precipitation	ND	Dialysis	(14)
PLP1, PLP2, PLP3, PLP4	Leaf	UAE, liquid-to-solid ratio of 41:1, 49°C, 40 min, ultrasonic power of 204 W, dehydrated ethanol precipitation	3.84%	Sevag method deproteinization, DEAE-52, Sephadex G-100 column chromatography	(34)
PFB-1-0	Leaf	HWE, 20 mL/g liquid-solid ratio, 100°C, methanol reflux, 80% EtOH precipitation	98 g/kg	DEAE-Toyopearl 650 M chromatography, tap water dialysis	(49)
*P.* leaf polysaccharide	Leaf	UAEE, enzymolysis time 40 min, enzymolysis temperature 20.4°C, cellulases quantity 2000 U/g, ultrasonic time 60 min, 80% EtOH precipitation	4.54%	ND	(50)
PFPS	Leaf	HWE, 20 mL/g liquid-solid ratio, 80°C, 3 h, 2 replicate extractions, 95% EtOH precipitation, Sevag method deproteinization, dialysis	3.57%	ND	(51)
*P. frutescens* leaf polysaccharide	Leaf	EAE, 40 mL/g liquid-solid ratio, 1771.85 U/g cellulase, enzyme action temperature 53.7°C, enzyme treatment time 36.2 min, EtOH precipitation	17.91 mg/g	ND	(33)
PFP-1, PFP-2, PFP-3, PFP-4	Leaf	Ethanol degreasing, HWE, 20 mL/g liquid-solid ratio, 95% EtOH precipitation	ND	DEAE-52 cellulose column chromatography, dialysis	(52)
PLP-0.1-I, PLP-0.2-I, PLP-0.3-I	Leaf	UAE, 35.3 mL/g liquid-solid ratio, 210 W, 66.7°C, 51.5 min	6.853 ± 0.321%	Macroporous adsorbent resin D101, DEAE 52, Sephadex G-200, column chromatography, Sevag method deproteinization	(20)
PLP	Leaf	UEAE, compound enzyme dosage of 5.54%, 15 mL/g liquid-solid ratio, 48 min, 40°C	7.326 ± 0.291%	ND	(20)
*P. frutescens* leaf polysaccharide	Leaf	HWE, 30 mL/g liquid-solid ratio, 80°C, 6 h; Firstly, soak in hot water at 70°C for 3 h, then, using MAE to extract, loading volume of 10 mL, microwave time of 30 s, microwave power of 800 W	2.21%; 3.99%	Activated carbon decolorization, Sevag method deproteinization, DEAE-cellulose column chromatography	(30)
PFSP-2-1	Seed	Petroleum ether degreasing, HWE, 20 mL/g liquid-solid ratio, 85°C, 2.5 h, 3 replicate extractions, 75% EtOH precipitation	3.42 ± 1.97%	Sevag method deproteinization, polyamide chromatography column method decolorization, DEAE 52, Sephacryl S-500 HR column chromatography	(53)
*P. frutescens* seed polysaccharide	Seed	WAE, 25 (mL/g) liquid-to-feed ratio, 480 W microwave power, 3 min microwave processing time, hot water extraction at 90°C for 3 h, Sevag method deproteinization, EtOH precipitation	9.06%	ND	(29)
*P. frutescens* polysaccharide	Seed	Petroleum ether de-oiling, HWE, 80°C, 4 h, 70% EtOH precipitation	ND	ND	(22)
PSMP-0.1-I; PSMP-0.2-I; PSMP-0.3-I	Seed meal	UAE, 26.1 mL/g liquid-solid ratio, 229 W, 42°C, 51.8 min	6.137 ± 0.062%	Macroporous adsorbent resin D101, DEAE 52, Sephadex G-200, column chromatography, Sevag method deproteinization	(20)
PSMP	Seed meal	UEAE, compound enzyme dosage of 6.60%, 24.7 mL/g liquid-solid ratio, 60.5 min, 62°C	7.611 ± 0.20%	ND	(20)
PSMP-1	Seed meal	UAEE, 6.6% compound enzyme, 25 mL/g liquid-solid ratio, 62°C, 61 min, 80% ethanol (EtOH) precipitation	7.711 ± 0.201%	D101 resin adsorption, Sevag method deproteinization, DEAE-52 column chromatography	(35)
PSMP	Seed meal	*N*-hexane degreasing, UAE, liquid–solid ratio of 26.00 mL/g, 43°C, 52.00 min, 229.00 W ultrasonic power	6.137 ± 0.062%	ND	(18)
PFSP-1; PFSP-2; PFSP-3	Seed meal	Petroleum ether, degreasing 6 h, HWE, 20 mL/g liquid-solid ratio, 85°C, 2 h, 3 times, EtOH precipitation, Sevag method deproteinization	8.38%	DEAE-52 Cellulose, Sephadex G-100 column chromatography	(17)

ND stands for not detected.

## 3 Structure of *Perilla frutescens* polysaccharides

Structural characterization of polysaccharides is important since the structure is closely related to biological activity (54). The key structural features of polysaccharides include their monosaccharide composition, molecular weight (M_w_), chemical components, and bonding information (55). Different plant parts (i.e., the roots, leaves, stems, or seeds), extraction methods, and isolation and purification steps can lead to variations in polysaccharide structure (56). The structural characteristics of polysaccharides derived from different parts of *P. frutescens* are summarized in Table 2.

**Table 2:** Structural characterization of polysaccharides from the different parts of *P. frutescens*.

Polysaccharide name	Source	Molecular weight	Monosaccharide composition	Structural characterization	Analysis technique	References
PFP	Leaf	1.18 × 106 Da	Rha, Ara, Gal, Glc, Xyl, and GalA in a molar ratio of 0.13: 0.55: 1.40: 1.00: 0.13: 0.22	α-L-Ara*f*-(1→, →6)-β-D-Gal*p*-(1→, →4)-α-D-Glc*p*-(1→, →1,4)-β-D-Xyl*p*(1→, →4)-α-Gal*p*A-(1→, →2,4)-α-L-Rha*p*-(1→	HPGPC, FT-IR, IC, NMR	(15)
PFB-1-0-ii	Leaf	10 kDa	Ara (9.6%), Xyl (0.2%), Man (39.8%), Gal (14.5%) and Glu (45.6%)	ND	HPLC, GLC	(16)
PEPF	Leaf	ND	ND	ND	ND	(14)
PLP1, PLP2, PLP3, PLP4	Leaf	ND	ND	ND	ND	(34)
PFB-1-0	Leaf	ND	ND	ND	ND	(49)
*P.* leaf polysaccharide	Leaf	ND	ND	ND	ND	(50)
PFPS	Leaf	ND	ND	N-H(-CONH-), C-O-C glycosidic bonds	FT-IR	(51)
*P. frutescens* leaf polysaccharide	Leaf	ND	ND	ND	ND	(33)
PFP-1, PFP-2, PFP-3, PFP-4	Leaf	ND; 2.2 kDa; 5.2 kDa; 1.6 kDa	Man, Glu, Gal, Ara in a ratio of 51.3:32.1; Man, Rha, GluA, GalA, Glu, Gal, Ara, fructose (Fuc) in a ratio of 1.7:7.2:1.2:65.9:0.8:11.3:10.7:1.1; Man, Rha, GluA, GalA, Glu, Gal, Ara, Fuc in a ratio of 5.8:15.7:2.9:33.1:2.3:24.7:14.5:0.9; Man, Rha, GluA, GalA, Glu, Gal, Ara, Fuc in a ratio of 4.8:5.7:5.6:8.3:14.4:34.9:23.8:2.5	ND	HPGPC, HPLC	(52)
PLP-0.1-I, PLP-0.2-I, PLP-0.3-I	Leaf	ND	Rha, Fuc, Ara, Xyl, Man, Glu, Gal in a molar ratio of 22.88, 2.20:14.41:1.00:3.88:4.47: 24.55; Rha, ribose (Rib), Fuc, Ara, Man, Glu, Gal in a molar ratio of 20.44:1.00:1.98:6.55:4.58:15.45:21.52; Rha, Rib, Fuc, Ara, Xyl, GluA, Man, Glu, Gal in a molar ratio of 27.55:2.91:5.07:16.04:4.03:1.00:18.66:39.62:64.26	β-D-Glucopyranose	GC-MS, FT-IR	(20)
PLP	Leaf	ND	ND	ND	ND	(20)
*P. frutescens* leaf polysaccharide	Leaf	ND	ND	ND	ND	(30)
PFSP-2-1	Seed	8.81 × 106 Da	Ara, Gal, Glu, Xyl, GluA in a molar ratio of 20.207:11.223:1.228:18.232:0.331	The main chain is →1)-Ara*f*-(5→1)-Gal*p*-(6→1)-Gal*p*-(6→1)-Ara*f*-(5→1)-Ara*f*-(5→1)-Gal*p*-(6→1)-Ara*f*-(5→1)-Ara*f*-(5→1)-Araf-(3→1)-Xyl*p*-(4→ with side chains of →1,6)-Gal*p*-(3→1)-Ara*p*, →1,6)-Gal*p*-(3→1,3)-Gal*p*-(6→1)-Ara*f*, →1,6)-Gal*p*-(3→1)-Ara*p*, and →1,3)-Ara*f*-(5→1)-Glc*p*-(4→1)-Gal*p*-(3→1)-Glc*p*	HPGPC, IC, FT-IR, methylation analysis, NMR	(53)
*P. frutescens* seed polysaccharide	Seed	ND	ND	ND	ND	(29)
*P. frutescens* polysaccharide	Seed	ND	ND	ND	ND	(22)
PSMP-0.1-I; PSMP-0.2-I; PSMP-0.3-I	Seed meal	ND	Rha, Ara, Xyl, GalA, Man, Glu, Gal in a molar ratio of 1.31:22.98:5.60:2.72:3.21:1.00:12.44; Rha, Fuc, Ara, Xyl, GalA, Man, Glu, Gal in a molar ratio of 8.58:2.08:20.36: 6.39:3.63:1.00:6.10:18.49; Rha, Rib, Fuc, Ara, Xyl, GalA, Man, Glu, Gal in a molar ratio of 12.91:1.00:2.21:9.55:8.03:6.71:1.42:29.58:22.24	β-D-Glucopyranose	GC-MS, FT-IR	(20)
PSMP	Seed meal	ND	ND	ND	ND	(20)
PSMP-1	Seed meal	ND	Rha, Ara, Xyl, Man, Glu, and Gal in a molar ratio of 0.69: 11.13:5.55:0.90:1.00:4.71	β-D-glucopyranose	FT-IR, GC-MS	(35)
PSMP	Seed meal	ND	Rha, Ara, Xyl, Man, Glu, and Gal in the mass ratio of 3.196%: 43.901%:21.956%:4.244%:4.706%:21.997%	β-D-Xyl, α-L-Ara, β-D-Gal, β-L-Ara, (1→6) glycosidic, and (1→3/4) glycosidic	GC-MS, FT-IR, NMR	(18)
PFSP-1; PFSP-2; PFSP-3	Seed meal	1.06 × 105; 5.96 × 104; 3.72 × 104	Man, Gal, Xyl, Ara in a molar ratio of 0.01:0.06:0.11:0.81; Man, Xyl, Ara in a molar ratio of 0.28:0.28:0.41; Rha, glucuronic acid (GluA), Glu, Gal, Xly, Ara with a molar ratio of 0.013:0.024:0.040:0.080:0.120:0.700	Pyranose ring, C-O glycosidic bond	HPLC, FT-IR	(17)

### 3.1 Monosaccharide composition

Monosaccharide composition is an essential foundation for the structural analysis of polysaccharides. The acid hydrolysis method is used to completely break the glycosidic bonds of polysaccharides; after hydrolysis, the polysaccharide sample is neutralized, filtered, and derivatized for analysis of monosaccharide composition (57). High-performance liquid chromatography (HPLC), gas chromatography-mass spectrometry (GC-MS), gas chromatography (GC), gas-liquid chromatography, and ion chromatography (IC) are common methods employed to measure the monosaccharide composition and molar ratio of polysaccharides (15, 16, 47, 48). Using GC-MS analysis, the monosaccharide composition of a PSMP was determined to comprise rhamnose (Rha), arabinose (Ara), xylose (Xyl), mannose (Man), glucose (Glu), and galactose (Gal) at a mass ratio of 3.196%: 43.901%: 21.956%: 4.244%: 4.706%: 21.997% (18). Using IC analysis, the PFP extracted from the leaves was reported to comprise six major monosaccharides, including Rha, Ara, Gal, Glu, Xyl, and galacturonic acid (GalA), with the Glu and Gal monosaccharides being the most abundant (15). Furthermore, analysis of *P. Frutescens* polysaccharides extracted from the leaves and purified with DEAE-Toyopearl 650 M and Sephadex G-100 column chromatography showed that the proportions of Ara, Xyl, and Gal were gradually reduced during the progression of purification, whereas the proportion of Man and Glu significantly increased, which ultimately accounted for the major portion of the obtained *P. frutescens* leaf polysaccharides (16). Subsequent GC analysis showed that the PFB-1-0 fraction consisted of Ara (8.9%), Xyl (0.9%), Man (25.9%), Glu (16.8%), and Gal (24.9%) after DEAE-Toyopearl 650 M column chromatography purification, and the PFB-1-0-ii fraction consisted of Ara (9.6%), Xyl (0.2%), Man (39.8%), Glu (45.6%), and Gal (14.5%) after Sephadex G-100 column chromatography purification.

### 3.2 Molecular weight

The *M*_w_ not only affects the physical properties of polysaccharides, such as viscosity and solubility, but also affects their biological activities, forming the basis for characterizing the properties of polysaccharides (58). To date, the *M*_w_ of *P. Frutescens* polysaccharide has mainly been analyzed using HPLC and high-performance gel-permeation chromatography (HPGPC) detection techniques (16, 23, 51). Based on the available literature, the *M*_w_ of *P. frutescens* polysaccharide ranges from approximately 1.6 × 103 Da to 1.18 × 106 Da. For example, PFB-1-0-ii obtained from extraction of *P. frutescens* leaves using HWE and ethanol precipitation, followed by further separation and purification on a DEAE-Toyopearl 650 M column and a Sephadex G-100 chromatographic column, had an *M*_w_ of 10 kDa determined by HPGPC (16). By contrast, Niu et al. (52) obtained three main polysaccharide fractions from *P. frutescens* leaves by HWE, ethanol precipitation, and further purification on a DEAE-52 column with an *M*_w_ of 2.2 kDa and 1589.8 Da for PFP-2 and PFP-4, respectively, whereas PFP-3 was mainly composed of two segments with an *M*_w_ of 5.2 and 40.0 kDa, respectively, as determined by HPGPC. The difference in *M*_w_ among these *P. frutescens* polysaccharides is mainly attributed to variations in factors such as source, treatment method, and separation equipment. Similar variations in *M*_w_ have also been observed in studies on polysaccharides from Bupleuri Radix, and *Nelumbo nucifera* Gaertn. (lotus), and Radix Hedysari (26, 59, 60).

### 3.3 Chemical structures

**Figure 3:**
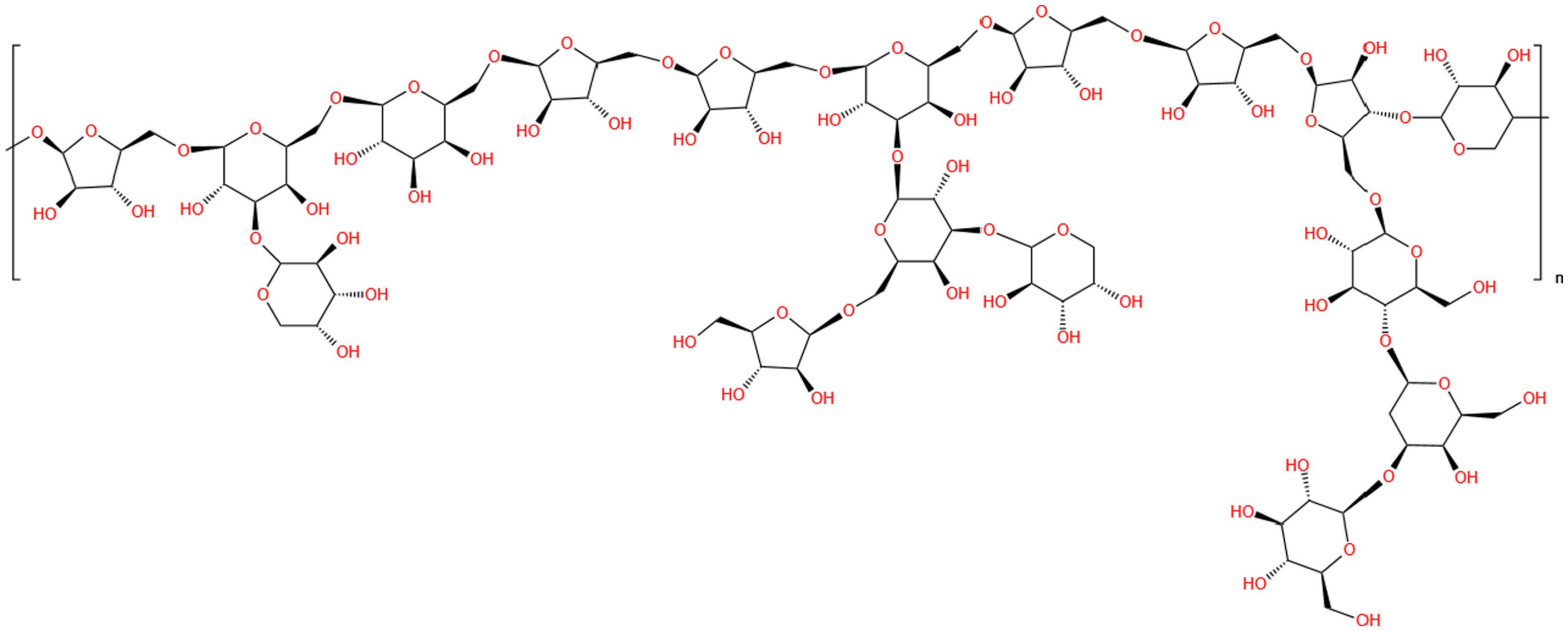
Diagram of the planar structure of *P. frutescens* polysaccharide (PFSP-2-1) (53).

The biological significance of polysaccharides is intimately associated with their complex structural properties and unique backbone features (47). Therefore, determining the chemical structure of polysaccharides is an important element in the investigation of their pharmacological effects and improvement of their applications (61). Chromatographic techniques, spectroscopic analysis, and other chemical analyses are effective methods for studying the structural characteristics of *P. frutescens* polysaccharides, including the types and linkages of sugar residues. According to the results of Fourier-transfer infrared spectroscopy (FT-IR), *P. frutescens* seed meal polysaccharide had five absorption peaks at 3,356 cm^−1^ (O−H), 2931 cm^−1^ (C−H), 1658 cm^−1^ (−C=O and −CHO), 1415 cm^−1^ (C−O), and 1,317 and 1,245 cm^−1^ (−COOH). The strong peaks noted at 1,072 and 1,047 cm^−1^ suggested the presence of galactopyranose and arabinofuranose in the backbone and branches of the sugar chain. GC-MS and one-dimensional nuclear magnetic resonance (NMR) spectrum (1H, 13C) analyses showed that *P. frutescens* seed meal polysaccharide was composed of β-d-Xyl, α-l-Ara, β-d-Gal, and β-l-Ara sugar residues, and was free of glucuronic acid (18). Moreover, Li et al. (53) obtained a homogenous polysaccharide faction of PFSP-2-1 from *P. frutescens* seed. Based on FT-IR, IC, methylation, and one-and two-dimensional NMR analysis, the structure of PFSP-2-1 showed a backbone of →1)-Ara*f*-(5→1)-Gal*p*-(6→1)-Gal*p*-(6→1)-Ara*f*-(5→1)-Ara*f*-(5→1)-Gal*p*-(6→1)-Ara*f*-(5→1)-Ara*f*-(5→1)-Araf-(3→1)-Xyl*p*-(4→; and three branches consisting of →1,6)-Gal*p*-(3→1)-Ara*p*, →1,6)-Gal*p*-(3→1,3)-Gal*p*-(6→1)-Ara*f*, →1,6)-Gal*p*-(3→1)-Ara*p*, and →1,3)-Ara*f*-(5→1)-Glc*p*-(4→1)-Gal*p*-(3→1)-Glc*p.* The link sites were at position C-3 of →3,6)-Gal*p*-(1→, C-3 of →3,6)-Gal*p*-(1→, and C-5 of →3,5)-Ara*f*-(1→, respectively (Figure 3). Using FT-IR, IC, and NMR spectroscopy, PFP was confirmed to be an acidic polysaccharide with a backbone comprising six sugar residues: α-l-Ara*f*-(1→, →6)-β-d-Gal*p*-(1→, →4)-α-d-Glc*p*-(1→, →1,4)-β-d-Xyl*p*-(1→, →4)-α-Gal*p*A-(1→, and →2,4)-α-l-Rha*p*-(1→ (15). However, the detailed chemical structures of polysaccharides from *P. frutescens* leaf, such as the linkage of sugar residues, have been not reported to date. Therefore, the detailed structures of *P. frutescens* polysaccharides from different sources and parts of the plant need to be analyzed more deeply and comprehensively to further understand the physicochemical and biological properties of *P. frutescens* polysaccharides.

### 3.4 Conformational features

**Figure 4:**
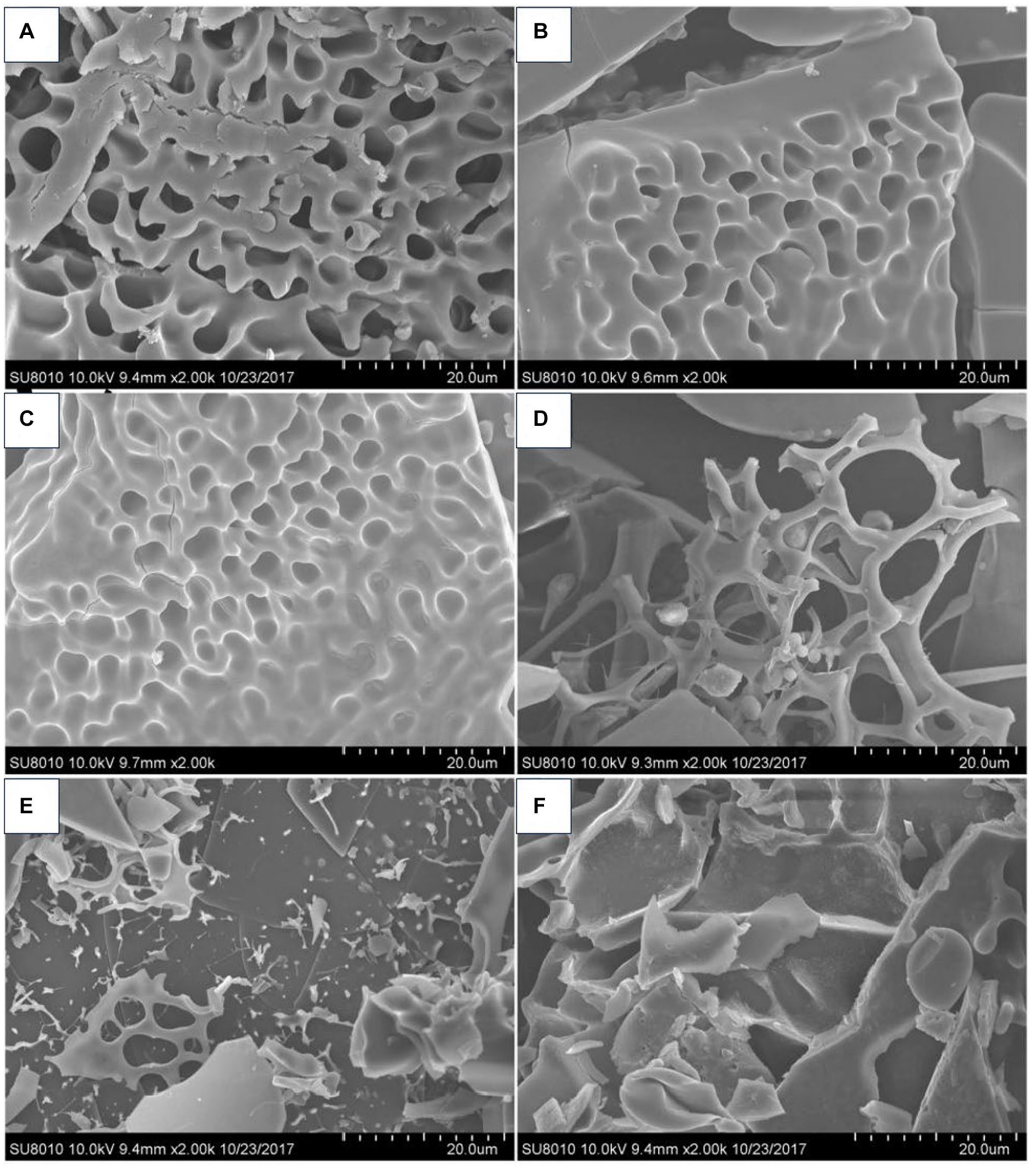
SEM micrographs of *P. frutescens* leaf polysaccharides (**A**: PLP-0.1-I; **C**; PLP-0.2-I; **E**: PLP-0.3-I) and *P. frutescens* seed meal polysaccharides (**B**: PSMP-0.1-I; **D**: PSMP-0.2-I; **F**: PSMP-0.3-I) (20).

Conformation refers to the three-dimensional structure created by the penetrating bonds formed by the molecular structure of the polymer and the physical force penetrating the space (62). Polysaccharides possess complicated substituents and diversified chemical structures, and their spatial conformation largely determines the various biological functions (63). Scanning electron microscopy (SEM) observations showed that the apparent morphology of polysaccharides obtained from *P. frutescens* leaves represents a combination of reticulated, lamellar, and chained forms, while the surface of the polysaccharides extracted from the *P. frutescens* seed meal is dominated by meshes and sheets (20) (Figure 4). Ding (23) carried out a comprehensive characterization of the morphological features of *P. frutescens* leaf polysaccharides via Congo red staining, circular dichroism, and SEM observations. The results revealed an overall orderly spatial structure of the *P. frutescens* polysaccharide, without a triple-helix structure, and the surface was smooth and flaky. Zhao et al. (51) similarly found a smooth lamellar structure on the surface of a *P. Frutescens* polysaccharide extracted from the leaves based on SEM observations.

## 4 Biological activities

**Figure 5:**
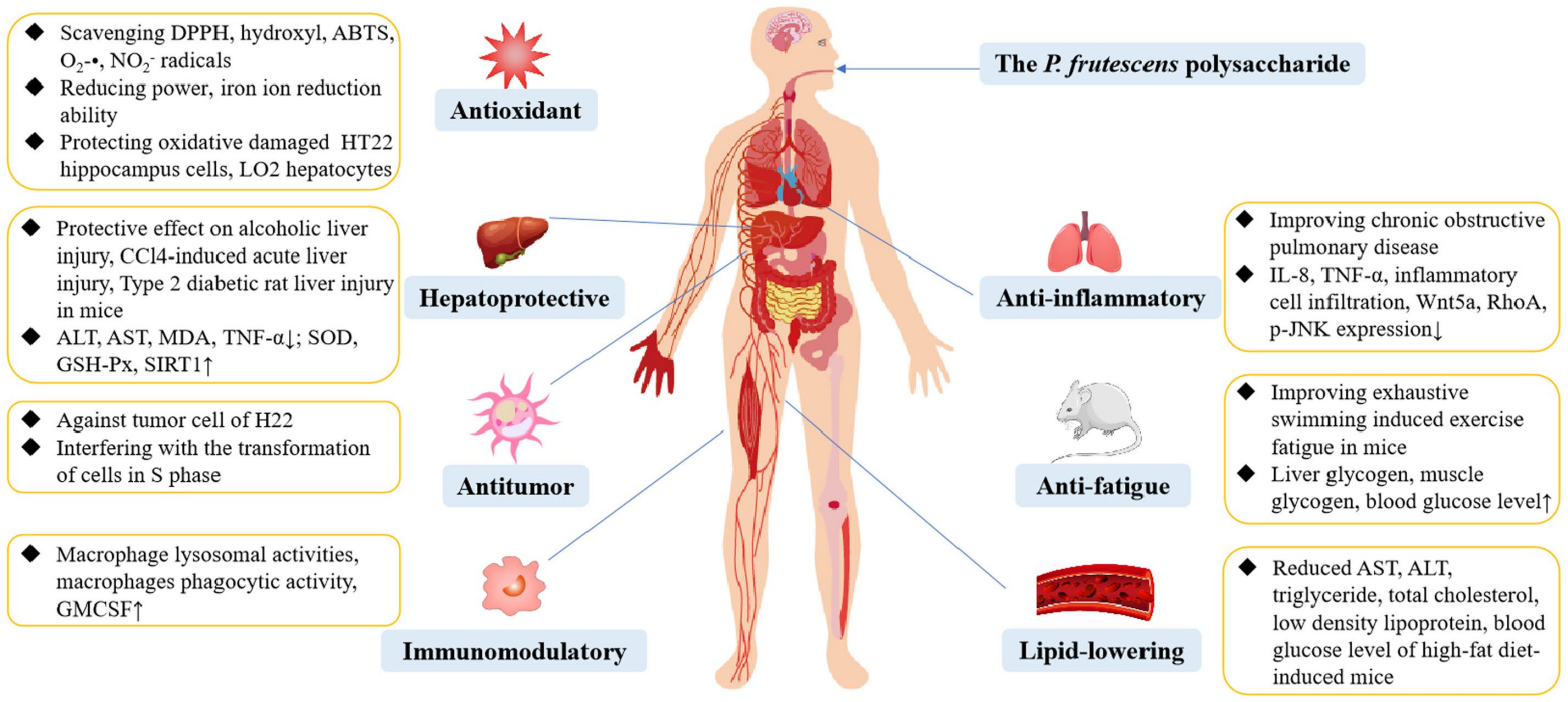
The biological activity of *P. frutescens* polysaccharide.

*P. frutescens* exhibits numerous edible and medicinal benefits, and its polysaccharide is an essential component conferring these characteristics. Indeed, *P. frutescens* polysaccharide possess a variety of biological activities, including antioxidant, antitumor, anti-fatigue, immunoregulation, hepatoprotective, anti-inflammatory, and lipid-lowering effects, which are summarized in Figure 5.

### 4.1 Antioxidant activity

Reactive oxygen species (ROS) are oxygen radicals in biological bodies, consisting of oxygen and oxygenated highly active molecules (e.g., superoxide anion, tissue peroxidation products, and free radicals). Typically, appropriate levels of ROS can facilitate immunity, repair, survival, and growth (64, 65). However, the excessive production of ROS will disrupt the dynamic redox balance, causing the human body to suffer damage, leading to multiple diseases (66). Hence, supplementation of antioxidants exogenously is essential for the body to fight or mitigate oxidative stress damage. *P. frutescens* polysaccharides demonstrate potential as effective antioxidants. The antioxidant activities of *P. frutescens* polysaccharides have been evaluated *in vitro* by examining the potential for scavenging 2,2-diphenyl-1-picrylhydrazyl (DPPH), 2,2′-azino-bis (3-ethylbenzothiazoline-6-sulfonic acid) diammonium salt (ABTS), hydroxyl (•OH), superoxide anion (O2-•), and nitrite ion (NO_2_-) radicals, along with iron-reduction capacity assays (14, 20, 37). For example, 1,000 μg/mL of a *P. frutescens* seed meal polysaccharide (PMP) showed scavenging rates on •OH, O2-•, NO_2_-, and DPPH radicals of 49.70 ± 2.10%, 21.76 ± 1.09%, 60.75 ± 2.19%, and 72.95 ± 4.19%, respectively (67). The scavenging ability of PMP for O2-• showed a certain dose-dependent effect at a polysaccharide mass concentration range of 62.5–1,000 μg/mL. However, Zhu et al. (68) showed that the scavenging rate of *P. frutescens* seed meal polysaccharide on O2-• radicals exhibited a trend of initial increase followed by a decrease; when the concentration of the polysaccharide reached 1,000 μg/mL, the formation of free radicals was promoted rather than suppressed. The reason for these discrepant effects could be attributed to the different structures and compositions of polysaccharides. Moreover, a PSMP exhibited a 73.83% scavenging rate of •OH radicals at 5,000 μg/mL and a 91.10% scavenging rate of ABTS radicals at 600 μg/mL (18). In general, the high antioxidant activity of polysaccharides is related to the enrichment of uronic acid groups (39). However, no uronic acid was detected in the PSMP, and the antioxidant properties were instead attributed to the presence of Man and Glu (18). Overproduction of ROS induced by oxidative stress also plays a key role in neuronal damage. In H_2_O_2_-induced neurotoxic cellular assays, *P. frutescens* polysaccharide reduced ROS production, lowered malondialdehyde (MDA) levels, increased intracellular superoxide dismutase (SOD) activity, and counteracted H_2_O_2_-induced neuronal cell death by activating the PI3K/AKT pathway and negatively regulating the mitogen-activated protein kinase (MAPK) and nuclear factor-kappa B (NF-κB) pathways in HT22 hippocampus cells (14).

### 4.2 Antitumor activity

Cancer remains a major global public health challenge. In the last few years, polysaccharides have attracted considerable attention in cancer research owing to their reported antitumor activity and low side effects (69). The antitumor effects of *P. Frutescens* polysaccharides could be attributed to their strong immunomodulatory abilities (15, 17). Treatment of H22 tumor-bearing mice with a *P. frutescens* leaf polysaccharide (PFP) at doses of 100 mg/kg and 300 mg/kg for three weeks significantly decreased tumor volume and weight and reduced thymic atrophy and splenomegaly in mice in a dose-dependent manner (15). *In vitro* investigations further showed that the PFP also inhibited tumor proliferation by blocking the S-phase of the cell cycle and decreasing mitochondrial membrane potential. The antitumor effect of PFP may be related to the Gal component. Treatment of tumor-bearing mice with *P. frutescens* seed polysaccharide (0.1, 0.3, and 0.5 mg for 10 days) significantly reduced the levels of lactate dehydrogenase, aldolase, and interleukin (IL)-10; increased the levels of IL-2 and tumor necrosis factor-alpha (TNF-α) in the serum of mice; and down-regulated the expression of the anti-apoptotic protein Bcl-2 and up-regulated the expression of the pro-apoptotic protein Bax (17). Collectively, these results indicated that *P. frutescens* seed polysaccharide can inhibit the growth of tumor cells *in vivo* by enhancing the autoimmunity of mice. Li et al. (53) found that a *P. frutescens* seed polysaccharide (PFSP-2-1) could significantly inhibit the growth of three types of hepatocellular carcinoma cells (HepG2, Hep3b, and SK-Hep-1), and the inhibitory effect gradually increased with the increase of PFSP-2-1 concentration. It has been hypothesized that Ara and Xyl in PFSP-2-1 are some of the factors inhibiting the growth of liver cancer cells. Moreover, the triple-helix high-level structure of PFSP-2-1 is also an important factor in its anti-tumor activity. In summary, *P. frutescens* polysaccharides exhibit excellent antitumor properties; however, the therapeutic effects will need to be further investigated on more cancer models along with determination of the conformational relationships.

### 4.3 Hepatoprotective effect

The liver is a crucial organ with essential functions in regulating metabolism, biotransformation, and detoxification (70). Factors such as autoimmune aggression of liver cells, medication misuse, alcohol consumption, infections with viruses, and cardiovascular disease predispose the liver to injury (71, 72). Several studies have demonstrated that *P. frutescens* polysaccharides have a hepatoprotective effect, which may be associated with their antioxidant and anti-inflammatory activities (19, 73). Li et al. (29) reported that *P. frutescens* seed meal polysaccharide administered at doses of 100, 300, and 500 mg/kg for 7 days alleviated CCl_4_-induced acute liver injury in an animal model. Specifically, the *P. frutescens* seed meal polysaccharide decreased serum aspartate aminotransferase (AST) and alanine aminotransferase (ALT) activities, as well as liver and spleen coefficients. In a type 2 diabetic rat liver injury model induced by high-fat and high-sugar feeding combined with intraperitoneal injection of streptozotocin, gavage of *P. frutescens* leaf polysaccharide at 0.15, 0.30, 0.60 g/kg for 28 days significantly reduced the levels of MDA, C-reactive protein, IL-6, TNF-α, and acetylated forkhead transcription factor protein in liver tissues, while the activities of glutathione peroxidase (GSH-Px), catalase (CAT), and SOD as well as the expression of sirtuin 1 (SIRT1) and FoxO1 proteins in liver tissues were significantly elevated (73). Thus, the *P. frutescens* leaf polysaccharide could improve type 2 diabetes-induced rat liver injury by inhibiting oxidative stress and inflammation, while regulating activation of the SIRT1/FoxO1 signaling pathway. Additionally, Tao et al. (19) found that *P. frutescens* leaf polysaccharide significantly improved hepatocyte inflammatory cell infiltration, bleeding, and liver steatosis in a mouse model of alcoholic liver injury disease (ALD) induced by chronic ethanol gavage. After gavage of the *P. frutescens* polysaccharides, the contents of IL-1β, IL-6, TNF-α, and MDA in the liver tissues of ALD mice were significantly decreased; the SOD and GSH-Px activities were significantly increased; and the relative expression levels of p-AMPKα/AMPKα and SIRT1 were significantly increased. These findings suggested that *P. frutescens* polysaccharide from the leaves might ameliorate liver injury in ALD model mice by modulating the activity of the SIRT1-AMPK signaling pathway in the liver tissues.

### 4.4 Immunomodulatory effect

Immunomodulation involves recognition of the body’s own and external substances, along with maintenance of the body’s physiological balance via the immune response to external antigens, which plays an essential role in the resistance to infections, tumors, and other diseases (31, 74). Natural polysaccharides play a vital role in immune regulation by stimulating immune cells such as T lymphocytes, B cells, macrophages, and cytotoxic T cells (75). To date, the immunomodulatory activity of *P. frutescens* polysaccharides has been demonstrated in a variety of *in vitro* and *in vivo* models. Kwon et al. (49) obtained a fraction from the crude polysaccharides extracted from *P. frutescens* leaves (PFB-1) by DEAE-Toyopearl 650 M chromatographic column elution named PFB-1-0, which significantly increased lysosomal enzyme activity as well as nitric oxide and TNF-α levels in mouse peritoneal macrophages. Moreover, PFB-1 could stimulate the production of IL-6 and granulocyte-macrophage colony-stimulating factors in mice. In a corroborative study, Kim et al. (16) showed that the active polysaccharide fractions (PFB-1, PFB-1-0, PFB-1-0-ii) from *P. frutescens* leaves at a concentration range of 1–100 μg/mL promoted macrophage lysosomal enzyme activity in a concentration-dependent manner. The immunomodulatory activity of these four *P. frutescens* leaf polysaccharides was attributed to the Man, Gal, and Glu components. Moreover, the high-molecular-weight *P. Frutescens* polysaccharide exhibited more effective macrophage-stimulating activity than the low-molecular-weight *P. Frutescens* polysaccharide. PFB-1-0-ii showed the best effect among all polysaccharide fractions, with 100 μg/mL PFB-1-0-ii increasing macrophage lysosomal enzyme activity by 245% in comparison to that of the untreated control. Together, these studies indicate that *P. Frutescens* polysaccharides have the potential to be developed into drugs and functional foods with novel immunostimulatory activities; however, the interaction mechanism between polysaccharides and the immune system remains unclear, warranting further research.

### 4.5 Other activities

In addition to the above biological activities, *P. Frutescens* polysaccharides also contribute to other health dimensions, including anti-fatigue, anti-inflammatory, and lipid-lowering effects. In a mouse model of fatigue induced by exhaustive swimming, gavage of *P. Frutescens* polysaccharides extracted from the leaves (5, 10, and 20 mg/kg for 4 weeks) significantly increased the forceful swimming time of mice, as well as the blood glucose level, liver glycogen content, and muscle glycogen content after exercise (76). These findings demonstrated a protective effect of the *P. Frutescens* polysaccharide in accelerating the removal of certain fatigue-causing metabolic substances, maintaining the normal physiological function of cells, and delaying fatigue. Wang et al. (77) tested the effectiveness of *P. Frutescens* leaf polysaccharide as an anti-inflammatory agent in a rat model of smoking combined with lipopolysaccharide-induced chronic obstructive pulmonary disease (COPD); treatment with 20 mg/kg *P. Frutescens* polysaccharide for 4 weeks *in vivo* resulted in improvement of lung function in COPD model rats. *P. Frutescens* polysaccharide treatment significantly decreased the expression levels of IL-8, TNF-α, Ras homologous protein A, Wnt5a, and p-JNK; reduced the thickness of bronchial wall and smooth muscle; and improved lung histopathologic changes. The inhibitory effect of *P. Frutescens* leaf polysaccharide on inflammation in COPD rats was mainly achieved by inhibiting the Wnt/PCP signaling pathway. In addition, Liu et al. (78) demonstrated the ameliorative effects of a *P. frutescens* seed polysaccharides (PFSP) on abnormal lipid metabolism and oxidative stress induced by a high-fat diet in mice. Supplementation of the PFSP (50, 100, and 200 mg/kg) in the feed for 8 weeks significantly decreased the levels of AST, ALT, triglyceride, total cholesterol, low-density lipoprotein, blood glucose, MDA, and the mRNA and protein expression levels of fatty acid synthesis-related genes. By contrast, PFSP supplementation increased the levels of high-density lipoprotein, fatty acid catabolism-related genes (*CPT-1, ATGL*) mRNA and protein expression, and the activities of SOD, GSH-Px, and CAT. Collectively, PFSP ameliorated high-fat diet-induced oxidative stress and fatty liver in mice. Based on the above research, the detailed biological effects or mechanisms of *P. Frutescens* polysaccharides are summarized in Table 2.

## 5 Conclusion and future prospects

*Perilla frutescens* is a highly promising edible and medicinal homologous resource; as the main components of *P. frutescens*, the polysaccharides of this herb have received increasing research attention in recent years. In this review, the extraction, purification, structural characterization, and associated biological activities of *P. Frutescens* polysaccharides have been summarized. Despite the remarkable advances achieved in *P. Frutescens* polysaccharide research, there are still several opportunities and challenges that remain to be resolved (Table 3).

**Table 3:** Summary of biological activities of polysaccharides from *P. frutescens* (“↓,” decrease; “↑,” increase).

Biological activities	Polysaccharide name	Types	Testing subjects	Doses/duration	Effects	Mechanisms	References
Antioxidant	PSMP	*In vitro*	DPPH, hydroxyl, ABTS radicals	2,000 μg/mL for DPPH radical, 5,000 μg/mL for hydroxyl radical, 600 μg/mL for ABTS radical	DPPH, hydroxyl, and ABTS scavenging rate was 75.00%, 73.83%, and 91.10%, respectively	ND	(18)
	PEPF	*In vitro*	DPPH, ABTS radicals, reducing power, ferric antioxidant power, H_2_O_2_-induced HT22 hippocampus cells	0.25–2 mg/mL in DPPH, and ABTS scavenging test, 0.25–2 mg/mL in reducing power and ferric antioxidant power assay, 500 μg/mL for oxidatively damaged HT22 hippocampus cells	Radical-scavenging activities, reducing power, and ferric antioxidant power↑; MDA, Bax, sub-G1 cells phase population↓; SOD, PARP, Bcl-2↑	Activating PI3K/AKT, negatively regulating the MAPK and NF-κB pathways	(14)
	PSMP-1	*In vitro*	DPPH, ABTS radicals	0.10–5.00 mg/mL in DPPH scavenging assay, 0.0625–2.00 mg/mL in ABTS scavenging assay	Scavenging DPPH and ABTS radicals, the IC_50_ was 2.078 ± 0.092 and 0.266 ± 0.009 mg/mL, respectively	ND	(35)
	PLP1, PLP2, PLP3, PLP4	*In vitro*	Reducing power, DPPH, hydroxyl, ABTS radicals	0.1–5.0 mg/mL for reducing power test, 0.1–5.0 mg/mL for DPPH, ABTS, hydroxyl radicals	DPPH, ABTS, hydroxyl radical-scavenging capacity and reducing power↑; antioxidant power of the four fractions was in the order of PLP3 > PLP2 > PLP1 > PLP4	ND	(34)
	PFP-1, PFP-2, PPFP-3, PFP-4	*In vitro*	DPPH, ABTS radicals, iron ion reduction ability	0.03–5.0 mg/mL for DPPH radical, 0.25–7.0 mg/mL for ABTS radical, 0.5–7.0 mg/mL in iron ion reduction ability assay	DPPH and ABTS scavenging capacity and ion reduction ability↑	ND	(52)
	*P. frutescens* leaf polysaccharide	*In vitro*	DPPH, hydroxyl radicals	0.1 mg/mL in DPPH scavenging test; 1.0 mg/mL in hydroxyl scavenging test	DPPH scavenging rate (94.17%) and hydroxyl scavenging rate (90.06%)	ND	(50)
	PMP	*In vitro*	Hydroxyl, O_2_-•, NO_2_-, DPPH radicals, H_2_O_2_-induced oxidative damaged LO2 hepatocytes	62.5–1,000 μg/mL for hydroxyl, O_2_-•, NO_2_-, and DPPH radicals; 200, 400, 800, 1,000 μg/mL for LO2 cell	Scavenging hydroxyl, O_2_-•, NO_2_-, and DPPH radicals, the EC_50_ was 964.59, 6376.84, 275.24, and 333.55 μg/mL, respectively; LO2 cell survival rate, lactic dehydrogenase, mitochondrial membrane potential, GSH, SOD, CAT, Bcl-2 expression↑; ROS, MDA, Bax expression, PARP expression↓	Regulation of the mitochondria-mediated apoptosis signaling pathway	(67)
	PLP-0.1-I, PLP-0.2-I, PLP-0.3-I, PSMP-0.1-I, PSMP-0.2-I, PSMP-0.3-I	*In vitro*	DPPH, hydroxyl, ABTS radicals	1–5,000 μg/mL for DPPH, hydroxyl, ABTS radicals, 1–500 μg/mL for reducing power assay	DPPH, hydroxyl, and ABTS radical-scavenging activities, and reducing power↑; ABTS scavenging ability was in the order of PLP-0.1-I > PLP-0.2-I > PLP-0.3-I > PSMP-0.1-I > PSMP-0.2-I > PSMP-0.3-I	ND	(20)
Antitumor	PFSP-2-1, PFSP-2-2	*In vitro*	HepG2, Hep3b, SK-Hep-1 cells	100, 200, 400, 800, and 1,600 μg/mL	Ability to inhibit the activity of HepG2, Hep3b, and SK-Hep-1 cells↑; HepG2, Hep3b, and SK-Hep-1 cells survival rates were 53.34%, 70.33%, 71.06% and 61.07%, 75.58%, 64.02% treating with 1600μg/mL of PFSP-2-1 and PFSP-2-2, respectively	ND	(53)
	PFP	*In vivo*	H22 hormonal mice	100 and 300 mg/kg for 3 weeks	Thymus index, tumor weight and volume, mitochondrial membrane potential↓; spleen index and tumor cell apoptosis↑	Interference with the transformation of cells in S phase	(15)
	PFSP-2	*In vivo*	H22 hormonal mice	0.1, 0.3, 0.5 mL (1 mg/mL), 10 days	LDH, aldolase, IL-10, Bcl-2 protein expression↓; IL-2, TNF-α, and proapoptotic protein Bax expression↑	Activates the ability of immune cells to produce cytokines	(79)
Hepatoprotective	*P. frutescens* leaf polysaccharide	*In vivo*	ALD mice	0.3, 0.6 g/kg for 60 days	Hepatic fat cell degeneration score, ALT, AST, TG, TC, LDL, IL-1*β*, IL-6, TNF-α, MDA, and SREBP1c relative expression↓; HDL, SOD, GSH-Px, p-AMPKα/AMPKα, and SIRT1 relative expression↑	Activation of the SIRT1-AMPK signaling pathway, reduction in oxidative stress and inflammation in the body	(19)
	*P. frutescens* leaf polysaccharide	*In vivo*	Type 2 diabetic rat liver injury model	0.15, 0.30, 0.60 g/kg for 28 days	Fasting blood-glucose, liver index, TG, TC, AST, ALT, MDA, CRP, IL-6, TNF-α, Ac-FoxO1 expression↓; body mass, GSH-Px, CAT, SOD, SIRT1, FoxO1 expression↑	Inhibition of oxidative stress, anti-inflammation, modulation of SIRT1/ FoxO1 signaling pathway activation	(73)
	*P. frutescens* seed polysaccharide	*In vivo*	CCl4-induced acute liver injury in mice	100, 300, 500 mg/kg for 7 days	ALT, AST, liver coefficient, spleen coefficient, and spotted necrotic lesions on the surface of the liver↓	ND	(29)
Immunomodulatory	PFB-0, PFB-1, PFB-1-0, PFB-1-0-ii	*In vitro*	Male ICR mice macrophages	1, 10, 100 μg/mL	Macrophage lysosomal relative activities↑; stimulatory effect of four components on macrophage lysosomal enzyme activity in the order PFB-1-0-ii > PFB-1-0 > PFB-1 > PFB-0	ND	(16)
	PFB-1-0, PFB-1	*In vitro, in vivo*	Murine peritoneal macrophages	100 μg/mL for in vitro test; 0.5, 0.75, 1.0 and 1.25 g/kg for specific pathogen free ICR mice for 8 days	Murine peritoneal macrophages lysosomal enzyme activity, mouse peritoneal macrophages phagocytic activity, nitric oxide, tumor necrosis factor, IL-6, and GMCSF↑	ND	(49)
Anti-fatigue	*P. frutescens* leaf polysaccharide	*In vivo*	5, 10, 20 mg/kg for 4 weeks	Exhaustive swimming induced exercise fatigue in mice	Liver glycogen, muscle glycogen, and exhaustion swimming time, blood glucose level↑	ND	(76)
Anti-inflammatory	*P. frutescens* leaf polysaccharide	*In vivo*	20 mg/kg for 4 weeks	Chronic obstructive pulmonary disease rat	Forced vital capacity, forced expiratory volume in one second, peak expiratory flow, and average alveolar number↑; IL-8, TNF-α, alveolar expansion, inflammatory cell infiltration, mean interalveolar lining, Wnt5a, RhoA, and p-JNK protein relative expression↓	Inhibition of the Wnt/PCP pathway	(77)
Lipid-lowering	PFSP	*In vivo*	50, 100, 200 mg/kg for 8 weeks	High-fat diet-induced mice	Liver index, AST, ALT, triglyceride, total cholesterol, low-density lipoprotein, blood glucose level, MDA, and *CPT-1* and *ATGL* mRNA expression↓; high-density lipoprotein, SOD, peroxidase, glutathione peroxide enzyme, and *FAS* mRNA expression↑	ND	(78)

ND stands for not detected.

First, studies have been carried out to extract *P. Frutescens* polysaccharides by HWE, MAE, UAE, enzyme-assisted extraction, and UAEE. There is still a great deal of scope for the development of other extraction methods. In particular, efforts should be focused on developing new environmentally friendly and high-yield extraction technologies. Second, the isolation and purification methods of *P. Frutescens* polysaccharides are currently limited to the laboratory level; thus, these methods should be further optimized to obtain reproducible and stable products that are suitable for industrial-scale production. Third, investigations on the structure of *P. Frutescens* polysaccharides have primarily focused on the monosaccharide composition and molecular weight. However, research is lacking on the more detailed structures of the sequence of monosaccharides, types, and positions of glycosidic bonds, and structural fragments. Various classical approaches, including FT-IR, NMR, X-ray diffraction, atomic force microscopy, and methylation analysis, can be applied to characterize the chemical structure of *P. Frutescens* polysaccharides. Fourth, to date, researchers have mostly focused on the antioxidant activity of *P. Frutescens* polysaccharides, with fewer studies on other bioactivities and their relationship with *P. Frutescens* polysaccharides structure. Therefore, it is imperative to establish more appropriate pharmacological models to investigate the additional biological activities of *P. Frutescens* polysaccharides and further study the structure-activity relationships of the polysaccharide, as well as the exact molecular mechanisms underlying the observed activities. Finally, research on *P. Frutescens* polysaccharides is still limited to theoretical studies in animal and *in vitro* experiments, with a notable lack of relevant clinical, toxicity, and pharmacokinetic studies. Developing and applying *P. Frutescens* polysaccharides as nutraceuticals or natural medicines is a huge challenge. Hence, a large number of clinical trials should be carried out to ensure the efficacy and safety of *P. Frutescens* polysaccharides toward creating novel products with *P. Frutescens* polysaccharides as the active ingredient.

In summary, *P. Frutescens* polysaccharides possess diverse biological functions, which offer extensive prospects in the fields of food and medicine; however, there is still a long way to go to achieve the transformation of *P. Frutescens* polysaccharides into practical functional foods, nutraceuticals, or natural medicines. We hope that more researchers will pay attention to PFP in the future and further investigate its precise structure, active mechanism, and clinical utilization, to offer a scientific foundation for its further development and utilization as functional foods and therapeutic drugs.

## Author contributions

LZ: Validation, Writing – original draft, Writing – review & editing. LG: Writing – review & editing. KW: Conceptualization, Writing – review & editing. CR: Data curation, Writing – review & editing. YG: Methodology, Writing – review & editing. JL: Investigation, Software, Writing – review & editing. SY: Project administration, Writing – review & editing. XZ: Formal analysis, Writing – review & editing. XY: Resources, Writing – review & editing. YZ: Methodology, Writing – review & editing. BL: Writing – review & editing. SL: Writing – review & editing.
